# Errata

**Published:** 1889-07

**Authors:** 


**Errata.**—In editorial, on page 30, fifth line from top, for "obtaine" read "obtains." On page 31, in twelfth line from bottom, for "one day," read "our day." On page 32, fifth line from top, for "where," read "were;" and add, after "association" the omitted words "to sue for damages, he". By this omission the sense is destroyed. Same page "F. S. Davis," should be, "N. S. Davis."

